# The role of FOXO3 and Irisin in evaluating posterior cruciate ligament and knee functional recovery after surgical treatment of tibial avulsion fracture of the posterior cruciate ligament insertion

**DOI:** 10.5937/jomb0-60053

**Published:** 2026-04-15

**Authors:** Wenbin Shen, Yao Xiao, Xin Yu, You Zhang

**Affiliations:** 1 Yiling People's Hospital of Yichang City, Department of Orthopaedics, Yichang, Hubei

**Keywords:** FOXO3, Irisin, fractures, posterior cruciate ligament, knee joint, FOXO3, irisin, prelomi, zadnji ukrsteni ligament, kolenski zglob

## Abstract

**Background:**

To explore the predictive value of serum forkhead box protein O3 (FOXO3) and Irisin in evaluating posterior cruciate ligament (PCL) and knee functional recovery after surgical treatment of tibial avulsion fractures (TAFs) of the PCL insertion (PCL-TAFs).

**Methods:**

In this prospective cohort study, we recruited 125 patients with PCL-TAFs (research group) and 111 healthy controls (control group) between October 2023 and December 2024. Serum FOXO3 and Irisin levels were detected at preoperative baseline and 6-month follow-up. Clinical evaluations included Lysholm scores, knee joint range of motion (ROM), posterior drawer testing, and International Knee Documentation Committee Subjective Knee Form (IKDC) scores. Pearson correlation and ROC analyses examined biomarker associations with functional recovery.

**Results:**

PCL-TAF patients exhibited higher preoperative FOXO3 levels than controls, which decreased following treatment (P &lt; 0.05). Conversely, Irisin levels were initially lower than controls but increased post-treatment (P &lt; 0.05). The predictive performance of combined FOXO3 and Irisin yielded 70.59% sensitivity and 94.51% specificity for detecting posterior drawer test positivity (AU C= 0.880). FOXO3 and Irisin together could identify severe dysfunction (IKDC III-IV) with 75.76% sensitivity and 77.17% specificity (AU C= 0.784).

**Conclusions:**

Serum FOXO3 and Irisin show clinical utility as potential biomarkers for functional recovery monitoring following PCL-TAF surgery, providing an objective molecular basis, as evidenced by ROC analysis, for the formulation of individualized rehabilitation plans.

## Abstract (sr)

**Uvod:**

Cilj rada je da se ispita vrednost serumskog proteina O3 (FOXO3) i irisina u proceni oporavka zadnjeg ukrstenog ligamenta (PCL) i funkcionalnog oporavka kolena nakon hirurskog lečenja preloma tibijalne avulzije (TAF) insercije PCL (PCL-TAFs).

**Metode:**

U ovoj prospektivnoj kohortnoj studiji, regrutovali smo 125 pacijenata sa PCL-TAF-ovima (istraživačka grupa) i 111 zdravih kontrola (kontrolna grupa) između oktobra 2023. i decembra 2024. godine. Nivoi serumskog FOXO3 i irisina su detektovani na preoperativnom početku i nakon 6 meseci praćenja. Kliničke evaluacije su obuhvatale Lisholmove skorove, obim pokreta zgloba kolena (ROM), testiranje zadnje fioke i rezultate Subjektivnog oblika kolena Međunarodnog komiteta za dokumentaciju kolena (IKDC). Pirsonova korelacija i ROC analize ispitale su povezanost biomarkera sa funkcionalnim oporavkom.

**Rezultati:**

Pacijenti sa PCL-TAF pokazali su vise preoperative nivoe FOXO3 u odnosu na kontrolnu grupu, koji su se smanjili nakon tretmana (P &lt; 0,05). Nasuprot tome, nivoi irisina su u početku bili niži nego u kontrolnoj grupi, ali su se povećali nakon tretmana (P &lt; 0,05). Prediktivna efikasnost kombinovanog FOXO3 i irisina dala je osetljivost od 70,59% i specifičnost od 94,51% za otkrivanje pozitivnosti testa zadnje fioke (AU C= 0,880). FOXO3 i irizin zajedno mogli su da identifikuju tesku disfunkciju (IKDC III-IV) sa osetljivošću od 75,76% i specifičnošću od 77,17% (AU C= 0,784).

**Zaključak:**

Serum FOXO3 i irizin pokazuju kliničku korisnost kao potencijalni biomarkeri za praćenje funkcionalnog oporavka nakon PCL-TAF operacije, pružajući objektivnu molekularnu osnovu, sto je dokazano ROC analizom, za formulaciju individualizovanih planova rehabilitacije.

## Introduction

The posterior cruciate ligament (PCL) is the core structure maintaining posterior knee joint stability. Injuries to it, especially tibial insertion avulsion fractures, account for nearly 5% to 10% of all sports-related traumas and high-energy injuries [1]. Compared to isolated ligament substance injuries, tibial insertion avulsion fractures involve the disruption of the bone-ligament complex continuity. Delayed or inadequate fixation can predispose patients to posterior knee instability, accelerated cartilage degeneration, and abnormal lower limb alignment, fostering traumatic arthritis [2]. The current mainstays of treatment include open reduction and screw/suture fixation as well as arthroscopic minimally invasive fixation procedures [3]. Regarding postoperative management, knee joint functional recovery is influenced by multiple factors such as fracture healing quality, soft tissue repair status, muscle atrophy degree, and inflammation regulation [4]. Conventional assessment techniques, including radiographic examination, magnetic resonance imaging (MRI) scans, and subjective scoring tools like the Lysholm and International Knee Documentation Committee Subjective Knee Form (IKDC) questionnaires, are limited to evaluating anatomical restoration or symptomatic feedback. These conventional techniques primarily assess anatomical restoration or symptomatic feedback but fail to track the underlying molecular-level repair mechanisms or critical biological processes involved in functional recovery. In contrast, serum biomarkers offer the potential for dynamic monitoring of these molecular events.

In recent years, the study of biomarkers has provided a new perspective for evaluating the injury and repair of the motor system. Musculoskeletal repair is known to involve multifaceted biological processes, including fibroblast activation, collagen synthesis, inflammation modulation, and muscle satellite cell proliferation. These pathophysiological changes can be indirectly monitored through the dynamic fluctuations of specific serum molecules [5]. Among them, the forkhead box protein O3 (FOXO3), as a transcription factor, can participate in tissue repair by regulating autophagy, apoptosis, and muscle catabolism [6]. Specifically, elevated FOXO3 can inhibit collagen synthesis via suppression of the Akt/mTOR pathway, potentially hindering tissue repair [7]. Irisin, derived from muscle, promotes muscle-bone interface repair by activating fibroblast proliferation and collagen deposition [8]. It achieves this, in part, by activating integrin-FAK signaling in fibroblasts, enhancing their adhesion and migration to injury sites [9] their specific role in the repair of musculoskeletal system injury has been increasingly concerned [10][11], their interplay in knee joint function recovery after surgical treatment of tibial avulsion fractures (TAFs) of the PCL insertion (PCL-TAFs) has not been reported. However, the synergistic mechanism of FOXO3 and Irisin in the repair of PCL bone-ligament complex injury remains unclear, and their predictive value for postoperative functional recovery has not been well studied.

To address the gap in dynamic molecular monitoring of PCL-TAF repair, this study innovatively examines serum FOXO3 (involved in repair and metabolic regulation) and Irisin (promotes tissue regeneration) to determine their relationship with postoperative knee functional recovery. The data will contribute to evidence-based, individualized rehabilitation for PCL-TAFs, improving long-term patient outcomes.

### Materials and methods

### Research design

This study is a prospective cohort study, enrolling patients who received PCL-TAF fixation in our hospital from October 2023 to December 2024. Inclusion criteria: age 18-65 years, any gender; PCL-TAF diagnosis (Meyers-McKeever-Zaricznyj type II-IV) by clinical and imaging examinations; first-time injury, with surgery performed within 3 weeks of injury; treatment with open reduction and screw/suture fixation or arthroscopic minimally invasive fixation at our hospital. Exclusion criteria: other knee ligament injuries; pre-existing knee osteoarthritis, rheumatoid arthritis, or other chronic joint diseases; hepatic or renal dysfunction, or hematologic disorders; pregnant or lactating women; mental illness or cognitive impairment resulting in the inability to cooperate with rehabilitation training; re-operation due to complications within 3 months post-surgery.

### Research subjects

The sample size calculation was performed using G-Power software (version 3.1). With parameters set for a one-tailed test (effect size=0.3, α = 0.05, power=0.95), the analysis indicated a minimum requirement of 111 participants. Anticipating possible participant withdrawal, we increased recruitment by 10%, ultimately including 125 individuals in the research group. For comparison purposes, we also enrolled 111 healthy controls during the same timeframe. The institutional ethics committee approved all study procedures, and written informed consent was obtained from all participants.

## Methods

### Surgical methods

All operations were completed by the same senior surgical team in our hospital, using standardized surgical anesthesia and postoperative rehabilitation schemes.

### Disease assessment

Six months post-treatment, patients underwent posterior drawer testing [12] to evaluate cruciate ligament rehabilitation. Tibial posterior displacement values were categorized as follows: ≤3 mm (negative, stable) and >3 mm (positive, unstable). Knee joint functional recovery was evaluated according to the International Knee Documentation Committee (IKDC) scoring system [13], with four severity grades: Grade I: normal joint function without restrictions; Grade II: near-normal function with minimal limitations not impacting daily activities; Grade III: functionally impaired with noticeable effects on daily living and sports activities; Grade IV: severely compromised function preventing normal activities and potentially requiring reoperation.

### Laboratory examination

Blood samples were collected from both the research (at preoperative baseline and 6-month postoperative follow-up) and control (upon hospital admission) groups for testing. Serum levels of FOXO3 and Irisin were quantified using commercially available enzyme-linked immunosorbent assay (ELISA) kits (FOXO3 Kit: Wuhan VCS Technology Co., LTD., ELA-E0762h; Irisin Kit: Wuhan EILerite Biotechnology Co., LTD., E-EL-H5735) according to the manufacturers' instructions. The kits' antibodies have been validated for specificity and sensitivity by the manufacturers. The blood samples were placed into ethylenediaminetetraacetic acid (EDTA) anticoagulation tubes and stood at 4 for 30 minutes, followed by serum isolation via centrifugation (3000 rpm, 10 minutes). 100 μL standard samples were pipetted into corresponding wells (1-6) of the precoated plate, with well 7 serving as a PBST-only blank control. Subsequently, 200 μL blocking solution was added to all wells for a one-hour incubation. After removing the blocking solution, 100 μL standards were loaded into standard wells and 100 μL serum samples into test wells, followed by a two-hour incubation. Next, biotin-labeled detection antibody (1: 1000) was added (100 μL/well) to incubate for 1 hour. Subsequent processing involved: (1) 30-minute incubation with 100 μL streptavidin-HRP complex (1: 200), (2) 15-minute TMB substrate (100 μL) reaction at room temperature in darkness, and (3) acid termination (50 μL 0.1M H2SO4) preceding immediate 450nm absorbance (optical density [OD] value) measurement with a microplate reader. Rigorous quality protocols were implemented, featuring ≤10% within-run coefficient variation, synchronous QC samples, and Levey-Jennings chart verification of ≤15% variation across 20 successive assays without observable trends. The intra-batch coefficient of variation (CV) was required to be ≤ 10%. Quality control samples were included in each batch, and Levey-Jennings control charts were generated. The acceptance criteria included a CV ≤15% across 20 consecutive runs, with no observable trend shifts.

### Statistical methods

Data were imported into SPSS 24.0 for statistical analysis. Chi-square tests were used for the comparison of counting data [n(%)]. Measurement data were subjected to Shapiro-Wilk testing. If a normal distribution was followed, the data were presented as (χ̄±s) and compared by independent sample t tests (comparison between groups) or paired t tests (comparison within groups). Non-normally distributed data [expressed as median (P25, P75)] were analyzed using the Mann-Whitney U test for between-group comparisons, while the Wilcoxon test was applied for within-group comparisons. ROC analysis quantified diagnostic utility, with area under the curve (AUC) values indicating discriminatory power. The best cut-off value was determined by Youden index. The influencing factors were analyzed using Logistic regression. OR>1 indicates a risk factor, OR<1 indicates a protective factor. Results achieving P < 0.05 were deemed statistically significant.

## Results

### Clinical data comparison

The analysis indicated no significant difference in age, sex, and BMI between the research and control groups (P > 0.05, Table 1), confirming group comparability.

**Table 1:** Comparison of clinical baseline data.

	Control group	Research group	t and χ^2^	P
	n = 111	n = 125		
Age (χ̄±s)	49.58±8.98	50.61±8.65	0.898	0.370
Sex [n(%)]			0.143	0705
male	72 (64.86)	84 (67.20)		
female	39 (35.14)	41 (32.80)		
BMI (kg/m^2^) (χ̄±s)	24.44±2.70	24.87±2.50	1.266	0.207
SBP (mmHg) (χ̄±s)	123.21±11.85	122.66±18.41	0.270	0.788
DBP (mmHg) (χ̄±s)	72.65±10.83	72.73±13.10	0.050	0.960
Combined diabetes mellitus [n(%)]			0.697	0.404
yes	20 (18.02)	28 (22.40)		
no	91 (81.98)	97 (77.60)		
Combined hypertension [n(%)]			0.082	0.775
yes	12 (10.81)	15 (12.00)		
no	99 (89.19)	110 (88.00)		
Time from injury to operation (d) (χ̄±s)		10.13±4.19		
Side of the disease [n(%)]				
left	-	74 (59.20)		
right	-	51 (40.80)		
Types of Meyers-McKeever-Zaricznyj [n(%)]				
II	-	82 (65.60)		
III	-	43 (34.40)		
Causes of injury [n(%)]				
Sports injuries	-	42 (33.60)		
Traffic injuries	-	60 (48.00)		
Other	-	23 (18.40)		

### Comparison of FOXO3 and Irisin expression

**Figure 1:**
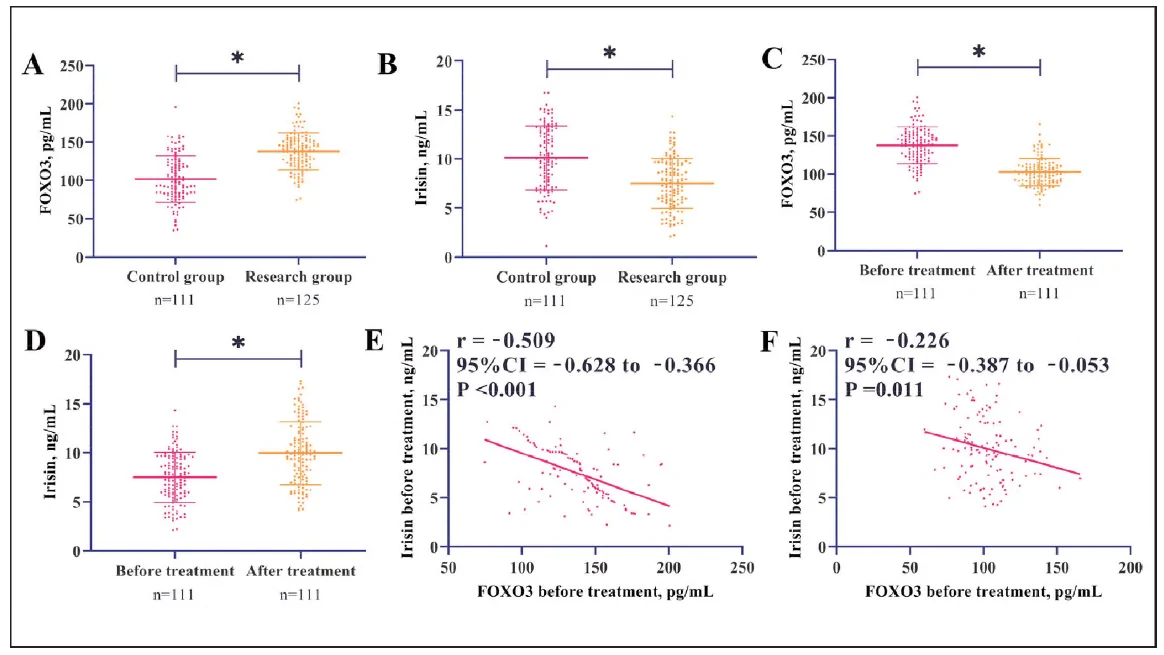
Expression of FOXO3 and Irisin in PCL-TAFs. A: Comparison of FOXO3 between control and research groups. B: Comparison of Irisin between control and research groups. C: Comparison of FOXO3 before and after treatment in the research group. D: Comparison of Irisin before and after treatment in the research group. E: Correlation analysis of FOXO3 and Irisin before treatment. F: Correlation analysis of FOXO3 and Irisin after treatment. *P < 0.05.

The pre-treatment FOXO3 and Irisin in the research group were (138.01±24.07) pg/mL and (7.50±2.55) ng/mL, respectively, with higher FOXO3 and lower Irisin compared to controls (P < 0.05). The results of regression analysis also confirmed that FOXO3 and Irisin were closely related to the occurrence of avulsion fracture of PCL insertion (Table 2). The treatment induced a reduction in FOXO3 and an elevation in Irisin in the research group (P < 0.05), with FOXO3 and Irisin demonstrating an obvious negative correlation (r=-0.509, -0.226, P < 0.05, Figure 1).

**Table 2:** Effect of FOXO3 and Irisin on PCL-TAFs.

Logistic	B	S.E.	Wals	P	OR	95%CI
FOXO3	0.042	0.007	40.430	< 0.001	1.043	1.030-1.059
Irisin	-0.217	0.057	14.274	< 0.001	0.805	0.719-0.901

### Evaluation of cruciate ligament recovery by FOXO3 and Irisin

**Figure 2:**
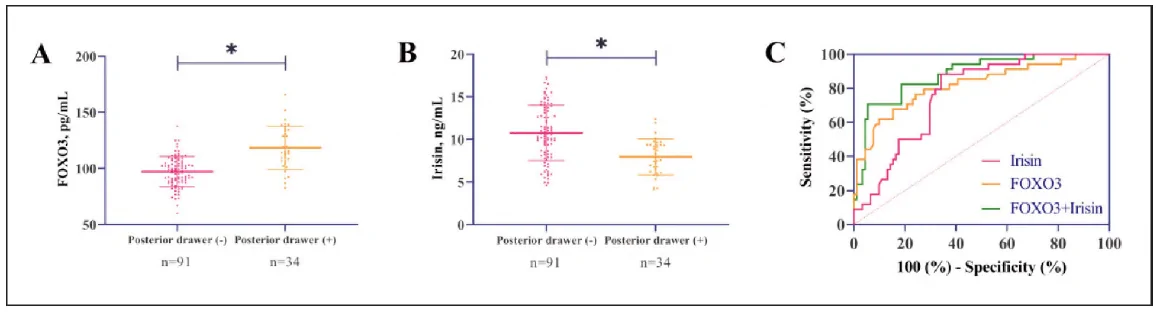
Relationship between FOXO3, Irisin and cruciate ligament recovery. A: Comparison of FOXO3 in patients with (+) and (-) posterior drawer test. B: Comparison of Irisin in patients with (+) and (-) posterior drawer test. C: Evaluation of cruciate ligament recovery by FOXO3 and Irisin.

Evaluation of posterior drawer test outcomes revealed that 91 patients (72.80%) tested negative. Negative cases demonstrated diminished FOXO3 and augmented Irisin compared to positive counterparts (P < 0.05, Figure 2A and Figure 2B). The AUC for detecting a positive drawer test using FOXO3 and Irisin alone was 0.819 and 0.765, respectively (Table 3). Subsequently, using regression analysis, we developed a formula for the combined detection of FOXO3 and Irisin (Table 3): joint detection = -7.014 + FOXO3x0.087 + IrisinX-0.366. The predictive performance of combined FOXO3 and Irisin yielded 70.59% sensitivity and 94.51% specificity for detecting posterior drawer test positivity (P < 0.05, Figure 2C). The AUC reached 0.880 (95%CI = 0.814-0.946), which was higher than that of a single diagnosis (Table 4).

**Table 3:** Relationship between FOXO3, Irisin and cruciate ligament recovery.

Logistic	B	S.E.	Wals	P	OR	95%CI
FOXO3	0.087	0.019	21.399	< 0.001	1.091	1.052-1.132
Irisin	-0.366	0.105	12.197	< 0.001	0.693	0.565-0.852

**Table 4:** Effect of FOXO3, Irisin in the evaluation of cruciate ligament recovery.

ROC	AUC	95%CI	Cut-off	Sensitivity (%)	Specificity (%)	P
FOXO3	0.816	0.729-0.908	>106.14	76.47	75.82	< 0.001
Irisin	0.765	0.682-0.849	<9.79	88.24	65.93	< 0.001
Joint detection	0.880	0.814-0.946	>0.527	70.59	94.51	< 0.001

### Evaluation of knee joint function by FOXO3 and Irisin

**Figure 3:**
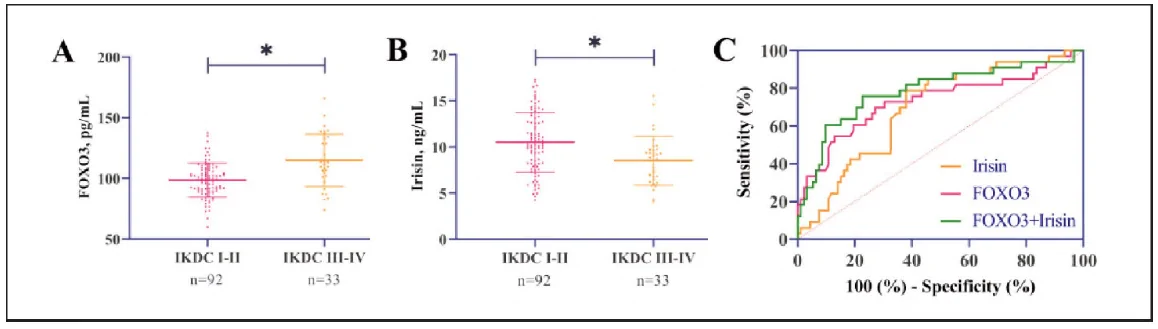
Relationship between FOXO3, Irisin, and knee function. A: Comparison of FOXO3 between IKDC grade III-IV patients and IKDC grade I-II patients. B: Comparison of Irisin between IKDC grade III-IV patients and IKDC grade I-II patients. C: Evaluation of knee joint function by FOXO3 and Irisin. *P < 0.05.

33 patients had IKDC grades III-IV and 92 patients had IKDC grades I-II. When comparing knee function, lower FOXO3 and higher Irisin were observed in IKDC I-II patients versus III-IV (P < 0.05, Figure 3A and Figure 3B). Regression analysis showed that FOXO3 and Irisin were closely related to the recovery status of knee function (Table 5). ROC analysis showed that FOXO3 and Irisin together could identify severe dysfunction (IKDC III-IV) with 75.76% sensitivity and 77.17% specificity (AUC = 0.784, 95%CI = 0.682-0.885) (P < 0.05, Figure 3C, Table 6), which was also significantly better than that of single test.

**Table 5:** Relationship between FOXO3, Irisin and knee functional recovery.

Logistic	B	S.E.	Wals	P	OR	95%CI
FOXO3	0.052	0.014	13.595	< 0.001	1.54	1.025-0.1084
Irisin	-0.188	0.081	5.396	0.020	0.829	0.708-0.971

**Table 6:** Effect of FOXO3, Irisin in evaluating knee functional recovery.

ROC	AUC	95%CI	Cut-off	Sensitivity (%)	Specificity (%)	P
FOXO3	0.734	0.622-0.846	>106.08	69.70	72.83	< 0.001
Irisin	0.693	0.593-0.793	<9.79	78.79	61.96	0.001
Joint detection	0.784	0.682-0.885	>0.280	75.76	77.17	< 0.001

## Discussion

Through prospective cohort analysis, this study found significantly elevated preoperative serum FOXO3 levels in PCL-TAF patients compared to healthy controls, accompanied by substantially reduced Irisin expression. After standardized surgical treatment and rehabilitation training, FOXO3 declined progressively while Irisin rose steadily over time, with their changing trends exhibiting close associations with Lysholm scores and ROM. Moreover, FOXO3 + Irisin detection showed strong predictive validity for postoperative cruciate ligament and knee joint dysfunction, offering valuable clinical utility for monitoring PCL-TAF rehabilitation progress.

As we all know, PCL-TAFs differ fundamentally from simple ligament rupture through their combined bone-ligament damage and consequent local microenvironmental dysregulation [14]. A critical manifestation is the RANKL/OPG imbalance at fracture sites, creating a pro-osteoclastic environment that drives excessive bone resorption [15]. Moreover, IL-1β and IL-6 released by inflammatory infiltrating cells (e.g., macrophages, neutrophils) inhibit muscle satellite cell differentiation through the JAK/STAT3 pathway [16]. This multi-factor interaction further complicates the repair process of PCL-TAFs. FOXO3 and Irisin, as key regulatory factors, may represent the dynamic equilibrium state of catabolism and anabolism, respectively. We hold that FOXO3 plays a dual role in PCL-TAFs: (1) Belonging to the forkhead box protein family, FOXO3 modulates various cellular processes including cell cycle inhibition, autophagic flux, and proteolysis through downstream gene regulation [17]. (2) Within musculoskeletal tissues, however, FOXO3 overactivation impedes muscle satellite cell expansion and promotes muscle fiber apoptosis, leading to muscle atrophy and hindered repair [18]. In this study, the increased preoperative FOXO3 levels could stem from fracture-induced inflammatory activation, where inflammatory mediators promote FOXO3 nuclear translocation through PI3K/Akt pathway stimulation, ultimately upregulating autophagy-related gene expression [19]. However, sustained FOXO3 elevation appears detrimental, with evidence demonstrating its capacity to hinder collagen production via Akt/mTOR pathway inhibition, compromising muscle-bone interface healing [7]. It is worth noting that the postoperative decrease in FOXO3 is synchronized with the improvement of Lysholm scores, indicating its potential role as a negative regulator reflecting inflammatory resolution and tissue repair initiation.

As a muscle-derived cytokine generated via FNDC5 cleavage into the bloodstream, Irisin can directly act on fibroblasts and bone marrow mesenchymal stem cells, thus facilitating type I collagen synthesis and extracellular matrix remodeling [20]. An animal experiment by He Z et al. [9] showed that Irisin-deficient mice showed delayed repair and decreased mechanical strength after ligament injury, while exogenous Irisin supplementation promoted organized collagen fiber alignment. Our findings reveal significant correlations between postoperative irisin elevation and improved cruciate ligament/knee function, suggesting potential mechanisms including: ① activating integrin-FAK signaling to enhance fibroblast adhesion/migration [21]; ② upregulating TGF-β1 expression to facilitate scar tissue formation [22]; and ③ suppressing MMP-9 activity to reduce collagen degradation [23]. Notably, a significant inverse connection between Irisin and FOXO3 was identified in this study, suggesting a potential antagonistic relationship—Irisin may block FOXO3's pro-apoptotic effects by inhibiting its nuclear translocation, forming a coordinated molecular network during tissue repair.

On the other hand, this study establishes the clinical utility of dual FOXO3/Irisin monitoring in assessing ligament/knee recovery, addressing critical gaps in current evaluation systems. While traditional imaging (X-rays, MRI) can visualize anatomical reduction and ligament continuity, they fail to quantify biomechanical properties or functional adaptation of repaired tissues. As Helito PVP et al. documented, ligament signal abnormalities on MRI may persist beyond clinical recovery timelines [24]. In contrast, serum FOXO3 and Irisin detection enables dynamic monitoring of the molecular progression of tissue repair, with their temporal changes offering molecular evidence for phase-specific rehabilitation planning. Although prior investigations have established biomarker roles in rehabilitation monitoring [25][26], single-marker approaches often suffer from limited biological specificity. The combined detection of FOXO3 and Irisin, on the other hand, significantly improves functional outcome prediction accuracy by capturing complementary catabolic (FOXO3) and anabolic (Irisin) metabolic states. This synergistic effect may be related to their reciprocal signaling pathway regulation - FOXO3 inhibits Irisin-mediated collagen synthesis, while Irisin feedback inhibits FOXO3's pro-apoptotic activity, forming a dynamic equilibrium window during tissue repair.

However, due to the relatively small sample size and single-center design, it is necessary to further expand sample recruitment to validate our conclusions. Besides, the lack of fracture classification-based subgroup analysis precludes the determination of the influences of fracture patterns on biomarker profiles. Furthermore, due to the failure to carry out animal experiments, the molecular mechanisms of FOXO3 and Irisin need to be further explored.

## Conclusion

Supported by their significant strong predictive performance in ROC analysis (AUC up to 0.784, 0.880), serum FOXO3 and Irisin can be used as potential biomarkers to evaluate knee joint functional recovery following PCL-TAF surgery. Their combined assessment not only reflects molecular homeostasis during repair but also provides an objective basis for the formulation of individualized rehabilitation programs. Future multicenter studies with larger cohorts are warranted to validate the clinical utility of this biomarker combination and to explore potential variations across different demographic or fracture severity subgroups to validate the clinical utility of this biomarker combination, concomitant with mechanistic research into FOXO3 and Irisin signaling pathways to identify targets for novel therapeutic interventions.

## Dodatak

### Availability of data and materials

The data that support the findings of this study are available from the corresponding author upon reasonable request.

### Funding

No funds, grants, or other support was received.

### Acknowledgements

Not applicable.

### Conflict of interest statement

All the authors declare that they have no conflict of interest in this work.

### Contribution

Wenbin Shen, Yao Xiao. These authors contributed equally to this work.
